# Broccoli-mediated gold nanoparticles: Eco-friendly synthesis and nano-bio interactions promoting wound healing and targeted cytotoxicity

**DOI:** 10.1016/j.jgeb.2025.100635

**Published:** 2025-12-12

**Authors:** Yasser M. Taay, Mustafa Taha Mohammed, Ali Hussain Alwan, Ahmad Hussein Ismail

**Affiliations:** a Department of Chemistry, College of Science, Mustansiriyah University, Baghdad, Iraq; b Iraqi Center for Cancer and Medical Genetics Research, Mustansiriyah University, Baghdad, Iraq

**Keywords:** AuNPs, Anticancer, Broccoli extract, HepG2 cell line, Wound healing

## Abstract

Biogenic gold nanoparticles (AuNPs) were synthesized using broccoli extract to assess their antioxidant activity, wound-healing potential, and selective anticancer effects. Green synthesis with broccoli offers an environmentally friendly way to produce stable and biocompatible nanomaterials. In this study, *Brassica oleracea* aqueous extract served as both the reducing and capping agent, producing AuNPs with a characteristic surface plasmon resonance peak at 560 nm and a well-defined cubic crystalline structure confirmed by XRD. TEM analysis showed uniformly dispersed, semi-spherical nanoparticles with an average size of 7.5 ± 3.6 nm. The biosynthesized AuNPs exhibited potent antioxidant activity, achieving 91.2 % DPPH scavenging at 100 µg/mL. *In vivo* tests demonstrated significantly faster wound healing, with approximately 90 % contraction by day 7 compared to 60 % in the control group, supported by histological evidence of increased collagen deposition and complete epithelialization. Moreover, AuNPs showed selective cytotoxicity towards HepG2 cancer cells (IC_50_ = 53.45 µg/mL) while maintaining over 70 % viability in normal HDF cells. This selectivity likely stems from differences in redox balance and mitochondrial vulnerability between cancer and healthy cells. Overall, the study presents broccoli-mediated AuNPs as multifunctional nanomaterials with potent antioxidant, regenerative, and targeted anticancer properties, highlighting their promising potential in biomedical applications.

## Highlights

• Green-synthesized AuNPs were successfully produced using broccoli extract.
• AuNPs accelerated wound healing and enhanced tissue regeneration *in vivo*.
• Histology confirmed improved epithelialization and collagen deposition.
• AuNPs showed selective cytotoxicity toward HepG2 with minimal effect on HDF.
• Biogenic AuNPs demonstrate promise as multifunctional therapeutic agents.

## Graphical abstract

**Figure:**
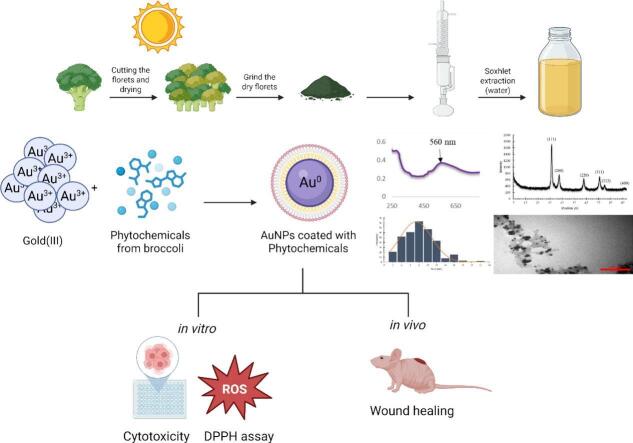
Schematic overview of the green synthesis of gold nanoparticles (AuNPs) using *Brassica oleracea* (broccoli) aqueous extract as a reducing and stabilizing agent. Phytochemicals in the extract reduce Au³⁺ ions to Au⁰, forming biogenic AuNPs characterized by UV–Vis (λ_max = 560 nm), XRD, and TEM analyses, which confirm a crystalline, nanoscale morphology. The biosynthesized AuNPs exhibited potent antioxidant and regenerative activity *in vivo*, promoting wound healing, while inducing selective cytotoxicity in HepG2 cancer cells.

## 1 Introduction

Nanotechnology has transformed biomedical science through the development of functional nanomaterials with adjustable physical, chemical, and biological properties.^1^, ^2^, ^3^, ^4^ Among these, gold nanoparticles (AuNPs) stand out due to their unique optical properties, chemical stability, and biocompatibility, making them ideal for applications in drug delivery, tissue engineering, and regenerative medicine.^5^, ^6^, ^7^ However, traditional physicochemical methods for synthesizing AuNPs often involve toxic reagents and require high energy, raising environmental and biomedical concerns.^8^, ^9^.

In response to these limitations, green synthesis strategies have emerged as sustainable alternatives that utilize natural biological systems, such as plant extracts, microorganisms, or biomolecules, as both reducing and stabilizing agents^10^. These environmentally friendly approaches not only mitigate environmental hazards but also introduce surface functionalities that improve biological compatibility and therapeutic effectiveness.^11^, ^12^ Plant-based synthesis is especially advantageous due to the abundance of secondary metabolites like flavonoids, phenolic acids, terpenoids, and alkaloids, which actively participate in the reduction of gold ions (Au^3+^) to elemental gold (Au⁰) and create natural capping layers that prevent nanoparticle aggregation.^9^, ^13^, ^14^, ^15^ Plant-derived phenolics and flavonoids function as electron donors that reduce Au^3+^ ions to metallic Au⁰, while also providing steric and electrostatic stabilization to prevent particle aggregation. This mechanism is widely reported in green-synthesis studies.^15^ .

*Brassica oleracea* (broccoli) belongs to the cruciferous family and is a well-known source of phytochemicals with potent antioxidant, anti-inflammatory, and anticancer properties.^16^ Its aqueous extract holds a variety of bioactive compounds, including sulforaphane, glucosinolates, polyphenols, and vitamins, that can serve as both reducing and stabilizing agents during nanoparticle formation.^17^, ^18^ Therefore, using broccoli extract in AuNP synthesis provides a dual advantage: an environmentally friendly production process and inherent therapeutic potential due to the bioactivity of the capping biomolecules.

In recent years, AuNPs have demonstrated significant promise in accelerating wound repair and modulating cellular responses in tissue regeneration^19^. Their small size and surface reactivity facilitate interaction with biological membranes, thereby promoting fibroblast proliferation, angiogenesis, and collagen deposition.^20^, ^21^, ^22^ Beyond their regenerative effects, AuNPs have also garnered significant attention for their potential anticancer activity, particularly against hepatic carcinoma models, such as HepG2 cells.^23^ The liver is a primary site for nanoparticle accumulation and detoxification, making hepatocellular systems an ideal model to evaluate nanotoxicological responses.^24^, ^25^ Biogenic AuNPs have been shown to induce apoptosis in cancer cells through the production of reactive oxygen species (ROS), mitochondrial impairment, and DNA damage, while causing minimal toxicity to normal fibroblasts.^26^, ^27^, ^28^ However, few studies have simultaneously examined the antioxidant, wound-healing, and selective anticancer effects of broccoli-mediated AuNPs, leaving a significant gap in understanding their multifunctional therapeutic potential.

The present work bridges two critical biomedical dimensions by investigating green-synthesized AuNPs using *Brassica oleracea* for their roles in wound healing and cytotoxicity testing. This combined approach gives a complete view of the therapeutic potential and safety of plant-made AuNPs, highlighting their importance in regenerative medicine and cancer therapy. The goal is to produce broccoli-derived biogenic AuNPs using an environmentally friendly aqueous extract and to test their antioxidant properties, wound-healing abilities, and targeted anticancer effects through both laboratory and animal studies.

## 2 Materials and methods

### 2.1 Preparation of Brassica oleracea var. Italica aqueous extract

Fresh florets of *Brassica oleracea* var. *italica* were harvested from farms in Wasit Governorate, Iraq, and confirmed taxonomically at the College of Agricultural Engineering Sciences, University of Baghdad. The plant material was carefully washed with distilled water to remove surface impurities and then air-dried in direct sunlight. The dried florets were finely ground into a smooth powder. Twenty grams of the dried powder were placed in a Soxhlet apparatus and extracted with 250 mL of distilled water for 6 h under continuous heating. The extract was filtered through Whatman No. 1 filter paper, and the pH was measured at 6.2 ± 0.1. The filtrate was concentrated to about one-quarter of its original volume and stored at 4 °C until use.

### 2.2 Synthesis of AuNPs

Gold nanoparticles were synthesized using the aqueous extract of *Brassica oleracea* var. *italica* as both the reducing and stabilizing (surfactant) agent. In a typical procedure, 50 mL of 4 mM chloroauric acid solution (HAuCl_4_, Merck, Germany) was gradually added to 50 mL of the broccoli extract at a controlled rate of 0.5 mL/min under ultrasonic irradiation using a Hielscher UP200St-T sonicator (Germany) operating at 26 kHz, 120 W, and 70 % amplitude. The temperature during sonication was maintained below 20 °C. A visible color change from pale yellow to purple occurred within 4–6 min, indicating nanoparticle formation.

**Fig. 1:**
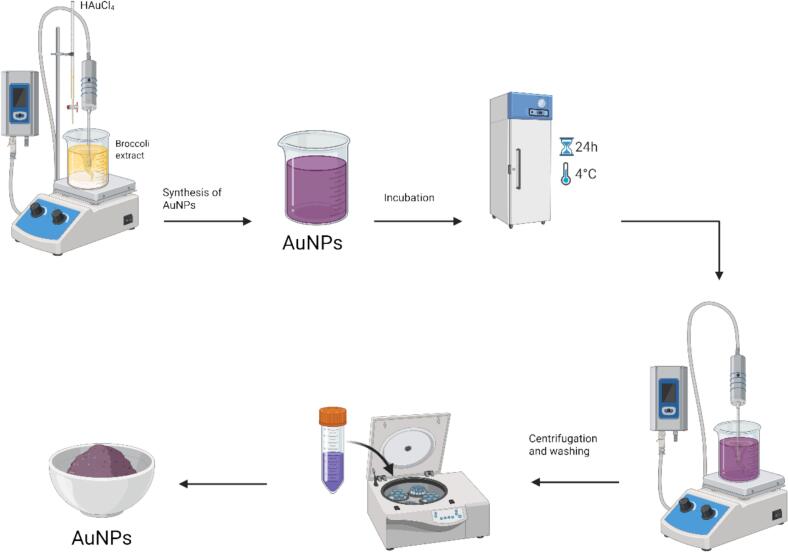
Schematic representation of the green synthesis of AuNPs using *Brassica oleracea* var. *italica* extract. The aqueous extract served as both a reducing and a stabilizing agent during the ultrasonic-assisted synthesis process. Chloroauric acid (HAuCl_4_) was added dropwise to the broccoli extract under sonication, followed by incubation at 4 °C for 24 h to allow nanoparticle formation. The colloidal suspension was then subjected to a secondary sonication and centrifugation to remove unreacted components, yielding purified AuNPs with stable dispersion. (For interpretation of the references to colour in this figure legend, the reader is referred to the web version of this article.)

Sonication was continued for 30 min after the complete addition of the gold precursor, then the colloidal solution was left undisturbed at 4 °C for 24 h to facilitate nanoparticle formation and stabilization. A second sonication was performed for 20 min under identical cooling conditions to ensure uniform dispersion. The resulting colloidal suspension was centrifuged at 4000 × g for 25 min, washed with deionized water, and then centrifuged again to remove unreacted components and residual biomolecules^29^ (Fig. 1).

### 2.3 Characterization of AuNPs

The optical properties of the synthesized AuNPs were analyzed using a UV–visible spectrophotometer (T80 PG Instruments, UK) to monitor the surface plasmon resonance (SPR) band, recorded at 1 nm wavelength intervals. Crystalline structure was evaluated with an XRD instrument (PHILIPS PW1730, The Netherlands) at 40 kV and 30 mA, using Cu–Kα radiation (λ = 1.5406 Å). Scans were conducted over 20–90° (2θ) with a step size of 0.02° and a scan rate of 2°/min, and the average crystallite size was determined using the Debye–Scherrer equation. The morphological features and particle size distribution of the AuNPs were examined by transmission electron microscopy (TEM, PHILIPS CM120, The Netherlands), providing insights into their shape, uniformity, and dispersion.

### 2.4 DPPH scavenging assay

The antioxidant potential of the synthesized AuNPs was assessed using the 2,2-diphenyl-1-picrylhydrazyl (DPPH) free radical scavenging assay. A 1.5 mM DPPH solution (Merck, Germany) was freshly prepared and mixed with various concentrations of AuNPs (10, 20, 40, 80, and 100 µg/mL) at a ratio of 1:2 (DPPH: AuNPs, v/v). A similar set of ascorbic acid concentrations was used as a standard antioxidant reference, while a methanolic DPPH solution served as the negative control. For comparison, broccoli extract was prepared at serial dilutions of 100 %, 80 %, 40 %, 20 %, and 10 % of the crude extract. All samples were incubated in the dark at room temperature for 30 min to prevent photo-induced degradation of DPPH radicals. The absorbance of each mixture was measured at 517 nm using a UV–visible spectrophotometer (Apel PD-303, Japan).^15^ The radical scavenging activity (%) was calculated according to Equation (1):

(1) $$\mathrm{Scavengingactivity}\%=\frac{A_{0}-A_{s}}{A_{0}}\times 100$$

Where A_0_ represents the absorbance of the control (DPPH), and A_S_ represents the absorbance of the samples.

### 2.5 Cytotoxicity

The *in vitro* cytotoxicity of the biosynthesized AuNPs was tested against HepG2 (human hepatocellular carcinoma) and HDF (human dermal fibroblast) cell lines. Both cell types were grown in Dulbecco’s Modified Eagle Medium (DMEM) supplemented with 10 % fetal bovine serum (FBS) and 1 % penicillin–streptomycin, and kept at 37 °C in a humidified incubator with 5 % CO_2_. Once the cells reach approximately 90 % confluence, they are detached using trypsin–EDTA, centrifuged at 5000 × g, and resuspended in fresh DMEM. For the cytotoxicity test, 12,000 cells per well are seeded into 96-well plates and incubated for 24 h to ensure proper attachment. Serial dilutions of AuNPs are prepared at concentrations of 1, 5, 10, 25, 50, 100, 250, and 500 µg/mL, with 100 µL of each dilution added to three replicate wells. Untreated wells serve as negative controls. After 48 h of incubation, the medium was aspirated, and the wells were gently washed with phosphate-buffered saline (PBS, 25 mM, pH 7.4). Then, 20 µL of MTT solution (5 mg/mL) was added to each well and incubated for 4 h to allow the formation of formazan crystals. The MTT solution was subsequently discarded, and 150 µL of dimethyl sulfoxide (DMSO) was added to dissolve the formazan. Absorbance was measured at 570 nm using a microplate reader,^30^ and cell viability (%) was calculated using Equation (2):

(2) $$\mathrm{Scavengingactivity}\%=\frac{A_{s}}{A_{0}}\times 100$$

Where A_S_ and A_0_ represent the absorbances of treated and untreated cells, respectively.

### 2.6 Wound healing

The wound-healing efficacy of biosynthesized AuNPs was investigated using male albino BALB/c mice aged 8–10 weeks. An AuNP-based ointment was formulated by dispersing 25 mg of AuNPs in 16 mL of olive oil, followed by mixing with 8 g of melted paraffin wax under continuous stirring until the mixture cooled and solidified. For comparative purposes, a broccoli-based ointment was prepared using the same procedure, but with lyophilized broccoli extract.^29^ .

The mice were randomly divided into three experimental groups (n = 5 per group):

(i) AuNPs-treated group,

(ii) Broccoli-treated group, and.

(iii) Control group (olive oil and paraffin wax only).

All animals were anesthetized with a ketamine–xylazine mixture (1:10), and their dorsal areas were shaved and disinfected. A full-thickness excision wound (2 cm in diameter) was created on the dorsal surface of each mouse. The respective ointments were applied topically once daily for eight consecutive days. Wound healing progress was monitored by taking digital photographs daily, and the percentage of wound contraction was measured using ImageJ software.

At the end of the treatment period, animals were humanely euthanized with an overdose of ketamine. Regenerated wound tissues were carefully excised and fixed in 10 % neutral-buffered formalin. The samples were processed using standard histological procedures, including graded alcohol dehydration, paraffin embedding, and sectioning. The tissue sections were then stained with hematoxylin and eosin (H&E) and examined microscopically to assess epithelial regeneration, collagen deposition, and inflammatory cell infiltration.^31^ .

### 2.7 Statistical analysis

Data from *in vitro* assays (DPPH and cytotoxicity) were obtained from three independent replicates (n = 3), while the *in vivo* wound healing experiment was performed with five animals per group (n = 5). All data are expressed as mean ± standard deviation (SD). Statistical analyses were conducted using SPSS version 26 (IBM Corp., USA). The Shapiro–Wilk test was used to assess data normality. Differences among groups were evaluated with one-way ANOVA followed by Tukey’s post hoc test. A significance level of p ≤ 0.05 was considered.

## 3 Results and Discussion

### 3.1 Characterization of AuNPs

**Fig. 2:**
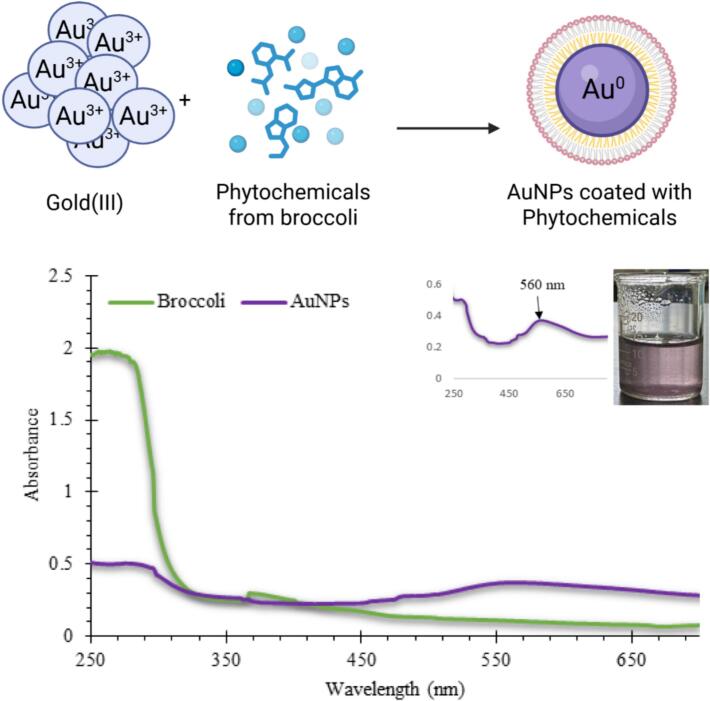
UV–visible spectral analysis confirming the formation of AuNPs synthesized using *Brassica oleracea* var. *italica* extract. The SPR peak at 560 nm indicates the successful reduction of Au^3+^ to Au⁰ and the formation of stable, phytochemical-capped AuNPs. The inset photograph shows the characteristic pink-purple coloration of the colloidal AuNP solution, while the schematic illustration depicts the interaction between broccoli-derived phytochemicals and gold ions during nanoparticle formation. (For interpretation of the references to colour in this figure legend, the reader is referred to the web version of this article.)

The UV–visible spectral analysis (Fig. 2) clearly showed the successful biosynthesis of AuNPs using *Brassica oleracea* extract. The distinct SPR band observed at 560 nm indicates the collective oscillation of surface electrons in response to light, a characteristic of nanoscale gold particles. The sharp, well-defined peak confirms that the phytochemicals in the broccoli extract reduced Au^3+^ ions to metallic Au⁰. The position and intensity of the SPR band indicate that the nanoparticles are spherical, monodisperse, and well-stabilized, as broad or shifted peaks usually signal aggregation or irregular shape.^32^, ^33^ The color change from pale yellow to purple further confirms the formation of AuNPs, consistent with the typical optical transition behavior of colloidal gold.^34^ .

The biomolecules in the broccoli extract, especially flavonoids, polyphenols, and glucosinolates, are probably responsible for both reducing and stabilizing the nanoparticles. These compounds donate electrons to reduce Au^3+^ ions to Au⁰ while also forming a capping layer that prevents particles from merging.^35^ This dual role is consistent with earlier research showing that phytochemicals from Brassica species serve as natural reductants and capping agents in nanogold production. The absorption maximum at 560 nm is slightly red-shifted compared to some reports of biosynthesized AuNPs (typically 520–550 nm),^36^, ^37^, ^38^ which may be due to variations in particle size, surface charge, or the capping biomolecules. Such spectral shifts are well-known indicators of differences in dielectric environment and particle–phytochemical interaction strength,^39^ further confirming the successful attachment of broccoli-derived biomolecules on the AuNP surface.

**Fig. 3:**
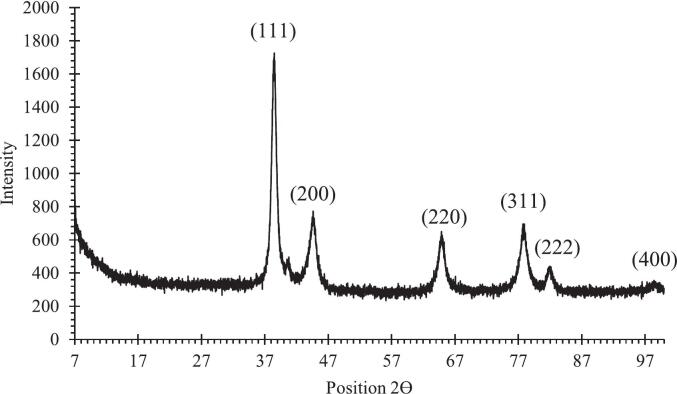
XRD pattern of AuNPs synthesized using *Brassica oleracea* extract, showing characteristic peaks at 2θ values of 38.32°, 44.54°, 64.82°, 77.87°, and 82.05° corresponding to the (111), (200), (220), (311), and (222) planes of fcc gold.

The confirmation of AuNP formation was verified by XRD analysis (Fig. 3). The diffraction peaks at 2θ = 38.321° (111), 44.542° (200), 64.820° (220), 77.877° (311), and 82.057° (222) correspond to the face-centered cubic (fcc) crystal structure of metallic gold (JCPDS No. 96–901-1613). An additional minor peak at 98.573° was attributed to the silicon substrate. The sharp and intense reflections indicate high crystallinity and purity of the synthesized AuNPs, with no detectable impurity phases. The average crystallite size, calculated with the Debye–Scherrer equation, was approximately 7.56 nm, confirming the formation of ultra-small nanocrystals. This small size indicates effective capping and nucleation control by the bioactive compounds in broccoli extract. Similar diffraction patterns have been reported for plant-mediated AuNPs synthesized using polyphenol-rich extracts, which typically exhibit fcc gold reflections within the range of 38–78°.^15^, ^37^, ^40^, ^41^ .

**Fig. 4:**
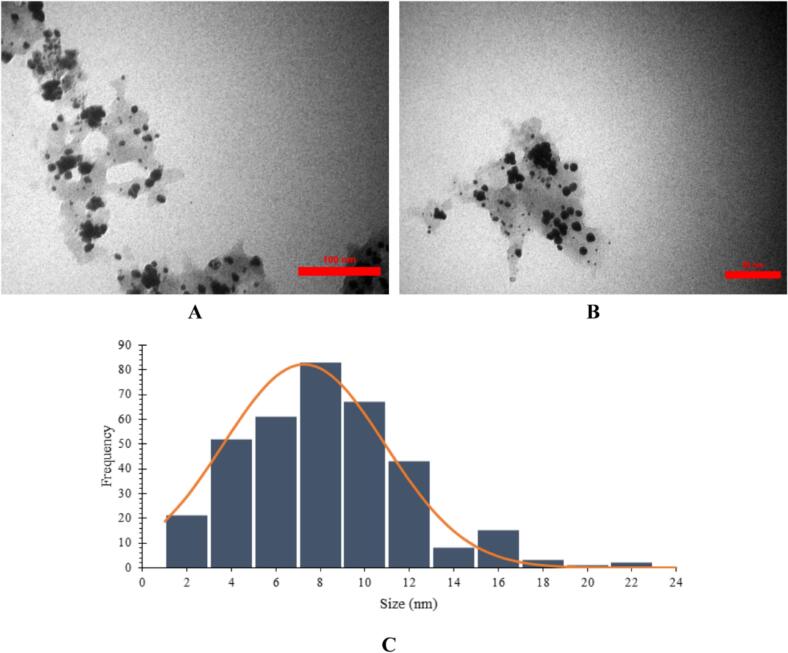
TEM images of the biosynthesized AuNPs using *Brassica oleracea* extract at different magnifications: **(A)** 100 nm and **(B)** 50 nm. The nanoparticles exhibit predominantly spherical morphology with uniform dispersion and minimal aggregation. **(C)** Particle size distribution histogram showing an average diameter of 7.5 ± 3.6 nm.

The morphology and particle size distribution of the biosynthesized AuNPs are shown in Fig. 4. The nanoparticles predominantly had semi-spherical shapes, with an average diameter of 7.59 ± 3.64 nm, and most were smaller than 12 nm in size. The narrow size range and limited clumping suggest that the phytochemicals in *Brassica oleracea* extract effectively acted as capping and stabilizing agents, controlling the nucleation and growth of AuNPs during reduction. This morphology aligns with previous studies on plant-mediated AuNPs, where biomolecules like flavonoids and phenolics play key roles in shaping crystal growth and preventing agglomeration.^42^, ^43^ .

### 3.2 DPPH scavenging activity of AuNPs

**Fig. 5:**
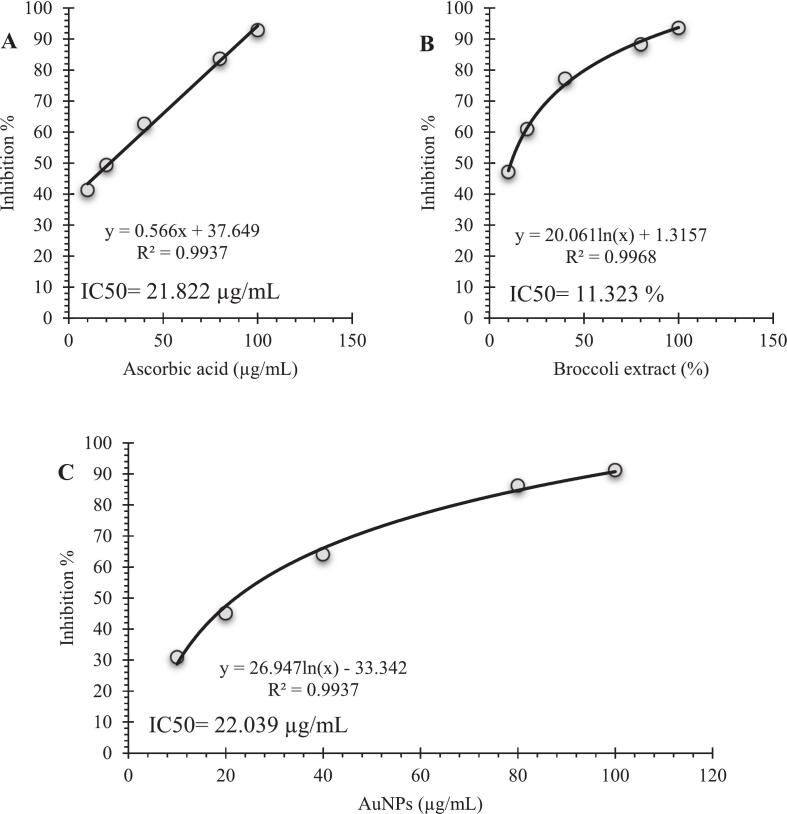
DPPH radical scavenging activity of (A) ascorbic acid, (B) *Brassica oleracea* extract, and (C) biosynthesized AuNPs. The IC_50_ values were 21.82 µg/mL, 11.32 %, and 22.04 µg/mL, respectively, indicating the strong antioxidant capacity of the broccoli extract and its derived AuNPs.

The antioxidant activity of the biosynthesized AuNPs, *Brassica oleracea* extract, and ascorbic acid was assessed using the DPPH radical scavenging method (Fig. 5). All tested samples showed a concentration-dependent scavenging effect. The calculated IC_50_ values were 11.32 % for the broccoli extract, 22.04 µg/mL for AuNPs, and 21.82 µg/mL for ascorbic acid. Because different measurement units were used for liquid extract (%) and nanoparticle/standard solutions (µg/mL), the comparison mainly indicates relative activity trends rather than exact equivalence.

The superior radical scavenging ability of the broccoli extract is due to its high content of antioxidant phytochemicals such as phenolic acids, flavonoids, glucosinolates, and vitamins, which effectively donate hydrogen atoms or electrons to neutralize DPPH radicals.^44^, ^45^ Although the AuNPs showed slightly lower activity than the crude extract, their IC_50_ value remained comparable to that of ascorbic acid, indicating that the capping layer maintained substantial redox potential. The moderate reduction in activity probably results from the consumption or structural modification of some reactive phytoconstituents during gold ion reduction. Nevertheless, the persistence of antioxidant capacity suggests that surface-bound phenolics on AuNPs remained active, providing sustained free-radical neutralization. These findings align with recent reports demonstrating that green-synthesized AuNPs possess antioxidant behavior derived from their surface phytochemicals.^46^, ^47^, ^48^, ^49^ .

Comparable antioxidant trends were observed for AuNPs synthesized with *Lens culinaris* extract, which showed potent DPPH scavenging and an IC_50_ similar to standard antioxidants like Butylated Hydroxytoluene.^50^ Similar results were also seen with nanoparticles prepared from *Rhus coriaria*, where the phenolic-rich surface layer notably enhanced radical neutralization.^51^ The consistency across these studies suggests that the antioxidant activity seen in our AuNPs is directly due to the phytochemical corona from *Brassica oleracea*, known to be rich in glucosinolates and polyphenols.

### 3.3 Wound healing activity

**Fig. 6:**
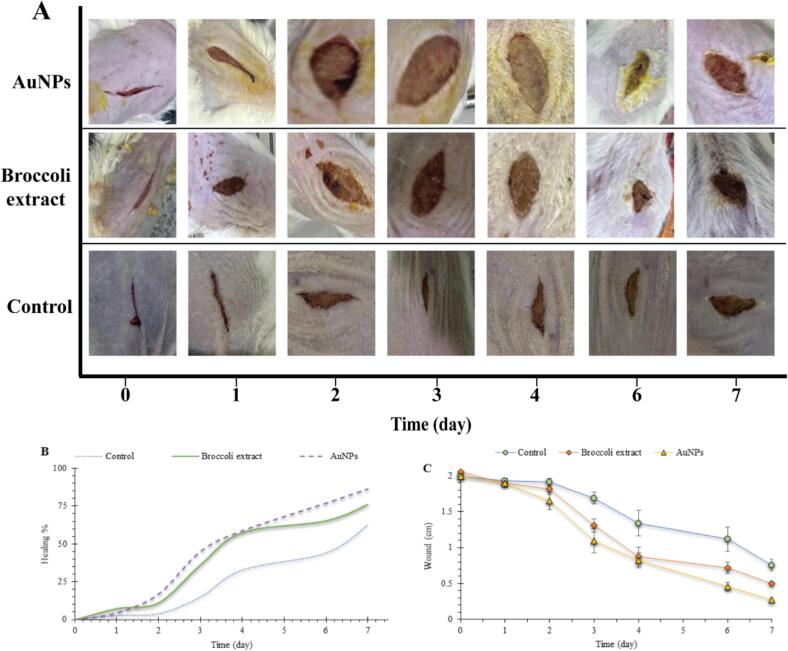
**(A)** Representative images showing the wound-healing process in BALB/C mice treated with AuNPs, broccoli extract, and control ointments over seven days. **(B)** Wound-healing percentage, and **(C)** wound size reduction across different groups. AuNPs showed a significantly faster healing rate compared to the broccoli extract and control groups (p < 0.05).

The wound-healing potential of the biosynthesized AuNPs was evaluated *in vivo* using the excision wound model in BALB/C mice (Fig. 6). The photographic sequence (Fig. 6A) displays progressive wound closure over seven days for the AuNPs, broccoli extract, and control groups. Visual observation revealed that both treatment groups exhibited faster tissue regeneration compared to the control, with the AuNPs-treated wounds demonstrating the fastest contraction rate and nearly complete epithelialization by day 7. Quantitative analysis of wound contraction (Fig. 6, Fig. 6) confirmed these results. The AuNPs group showed a significantly higher healing percentage (p < 0.05) than both the broccoli extract and control groups throughout the entire experimental period. By day seven, the wound size in the AuNPs group had decreased by 90 %, compared to 75 % in the broccoli group and 60 % in the control. The increased healing efficiency of AuNPs can be attributed to the synergistic effects of their nanoscale size, which enhances cellular uptake and interaction, as well as the bioactive capping agents from broccoli extract that provide antioxidant and anti-inflammatory properties.

**Fig. 7:**
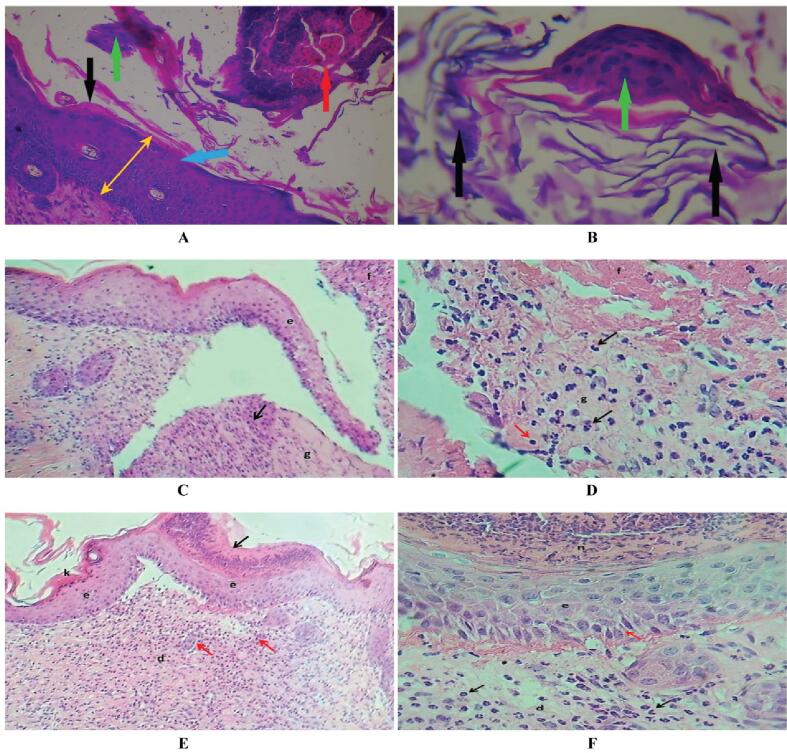
Representative histological sections of skin tissues (H&E stain) after 7 days of treatment showing differences in epithelialization and dermal regeneration among groups. **(A, B)** Control group: incomplete epithelialization (black arrows), presence of necrotic tissue (green arrows), and inflammatory cell infiltration (red arrows). The granulation tissue remains loosely organized (indicated by the yellow arrow). **(C, D)** Broccoli extract-treated group: partial epidermal regeneration (black arrows) and moderate fibroblast proliferation with early angiogenesis (red arrow). **(E, F)** AuNPs-treated group: complete and continuous epithelial layer (black arrow), thick epidermis with normal keratinization (k), aligned fibroblasts and dense collagen fibers (red arrows), and restored dermal–epidermal junction (d). Magnifications: A, C, E = 40×; B, D, F = 100 × . (For interpretation of the references to colour in this figure legend, the reader is referred to the web version of this article.)

The histological examination of the regenerated skin tissues, stained with H&E, revealed apparent morphological differences among the experimental groups (Fig. 7). In the control groups (A and B), the wound area exhibited incomplete epithelialization, with significant inflammatory cell infiltration and loosely organized granulation tissue. The epidermal layer was discontinuous, and fibroblast activity seemed weak, indicating delayed tissue repair. In contrast, the broccoli extract-treated group (C and D) showed moderate improvement, with partial epithelial regeneration and less inflammatory infiltration compared to the control. Newly formed capillaries and fibroblasts were visible within the dermal matrix, indicating the start of tissue remodeling; however, the epidermal layer remained thin and immature. The AuNPs-treated group (E and F) showed the most advanced healing process. A continuous, well-organized epithelial layer was present, along with dense collagen deposition and well-formed dermal structures. The epidermis appeared thicker with normal keratinization, and inflammatory cells were significantly reduced. The appearance of new blood vessels and aligned fibroblasts indicated active tissue remodeling and angiogenesis. These histological findings confirm the superior wound-healing ability of AuNPs compared to both the broccoli extract and control groups, demonstrating enhanced re-epithelialization, fibroblast proliferation, and extracellular matrix maturation.

During the wound healing process, oxidative stress has a dual role; moderate ROS levels promote cell proliferation and angiogenesis, while excessive ROS buildup results in chronic inflammation and delayed repair.^21^, ^52^, ^53^, ^54^ The scavenging ability of the synthesized AuNPs likely reduces ROS overproduction, maintaining a balanced redox environment that supports tissue regeneration. This antioxidant effect, along with the nanoscale size (7.6 nm) and high surface reactivity of AuNPs, improves cellular uptake and promotes fibroblast proliferation, collagen production, and epithelial migration.^55^ .

*In vivo* wound contraction analysis further confirmed the superior healing effectiveness of AuNPs compared to broccoli extract and control groups. The accelerated reduction in wound size and higher healing percentage in AuNP-treated mice can be attributed to improved angiogenesis and collagen remodeling. Histopathological examination supported these findings, showing complete epithelialization, dense collagen fiber deposition, and restoration of normal dermal structure in the AuNP group. In contrast, control tissues exhibited persistent inflammation and incomplete epithelial closure.^19^, ^56^, ^57^ For example, iron nanoparticles synthesized using *A. saralicum* significantly enhanced wound contraction, increased the number of fibroblasts and fibrocytes, and boosted collagen deposition. These effects are linked to the nanoparticles’ ability to reduce local oxidative stress and regulate early inflammation.^58^ These results closely match our histological findings of increased collagen deposition and complete re-epithelialization.

**Fig. 8:**
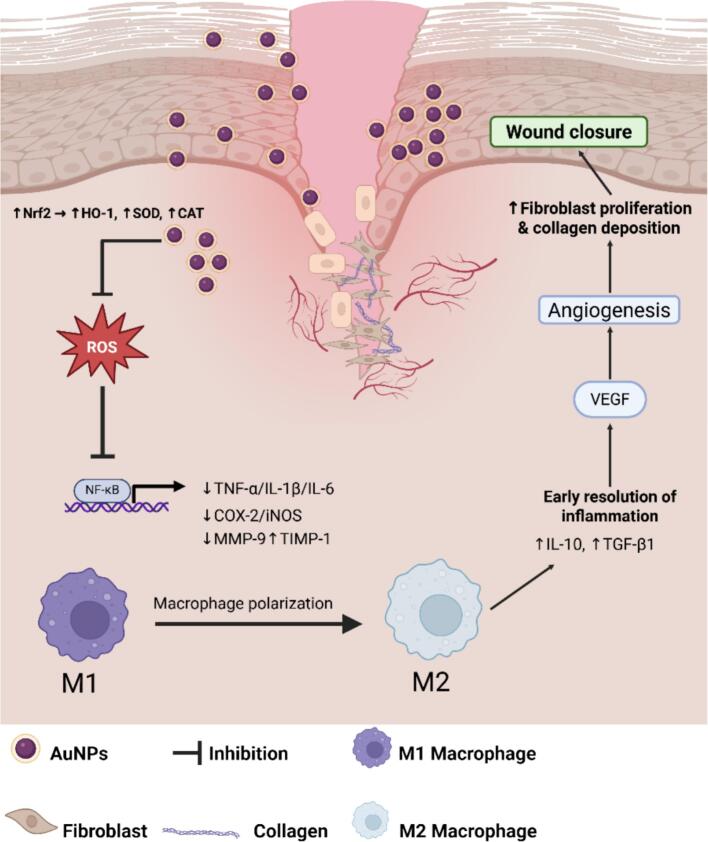
Proposed mechanistic illustration showing the wound-healing pathway mediated by broccoli-derived AuNPs. The AuNPs suppress reactive oxygen species (ROS) and inhibit NF-κB activation, leading to the downregulation of pro-inflammatory mediators (TNF-α, IL-1β, IL-6, COX-2, iNOS) and the matrix degradation enzyme MMP-9 while enhancing TIMP-1 expression. Simultaneously, AuNPs activate the Nrf2 antioxidant pathway (HO-1, SOD, and CAT), promoting redox balance and cellular protection. The reduction in inflammatory stress facilitates macrophage polarization from the M1 to M2 phenotype, accompanied by the release of IL-10 and TGF-β1, which drive fibroblast proliferation, collagen deposition, and angiogenesis through VEGF signaling. These coordinated cellular events culminate in accelerated re-epithelialization and wound closure, highlighting the dual anti-inflammatory and regenerative roles of biogenic AuNPs.

The proposed mechanism for these findings is shown in Fig. 8. Broccoli-mediated AuNPs likely mitigate oxidative stress by activating the Nrf2 signaling pathway, which in turn leads to elevated levels of antioxidant enzymes (HO-1, SOD, and CAT) and a decrease in ROS. This redox balance lessens NF-κB activation,^59^, ^60^ which results in lower levels of pro-inflammatory cytokines (TNF-α, IL-1β, IL-6) and inflammatory enzymes (COX-2, iNOS), thus shortening the inflammatory phase^56^. As a result, macrophages shift from the M1 to the M2 phenotype^61^, marked by higher release of IL-10 and TGF-β1^62^, which together support fibroblast growth, collagen production, and angiogenesis through VEGF signaling^19^, ^56^. These combined effects help resolve inflammation early and speed up wound closure,^63^, ^64^ showing that green-synthesized AuNPs act as multifunctional agents that provide antioxidant protection, reduce inflammation, and promote regeneration.

### 3.4 Cytotoxicity

**Fig. 9:**
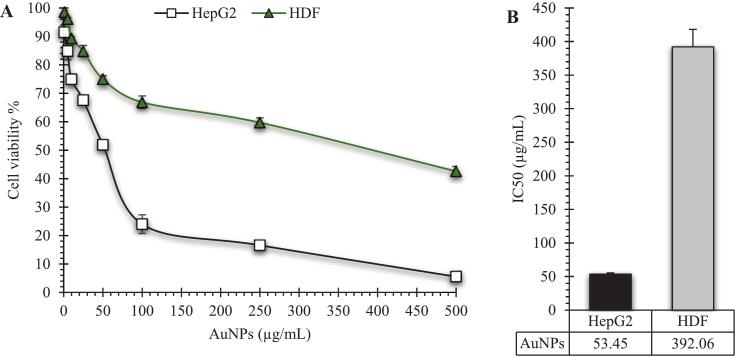
**(A)** Cell viability of HepG2 and HDF cell lines treated with increasing concentrations of broccoli-mediated AuNPs for 48 h. A concentration-dependent decrease in cell viability was observed, with HepG2 cells showing significantly higher sensitivity to AuNPs compared to HDF cells. **(B)** IC_50_ values of AuNPs against both cell lines, indicating selective cytotoxicity toward HepG2 (IC_50_=53.45 µg/mL) and significantly lower toxicity against HDF (IC_50_=392.06 µg/mL). Data are presented as mean ± SD (n = 3).

The cytotoxic effect of the biosynthesized AuNPs was evaluated against HepG2 and HDF cell lines. As shown in Fig. 9, AuNPs exhibited a concentration-dependent cytotoxic response, with a noticeable reduction in HepG2 cell viability at doses above 50 µg/mL. In contrast, HDF cells maintained relatively higher viability, even at higher concentrations of AuNPs. The IC_50_ values further confirmed this selectivity, with values of 53.45 µg/mL for HepG2 and 392.06 µg/mL for HDF (Fig. 9), indicating that the AuNPs possess vigorous anticancer activity while showing minimal toxicity toward normal fibroblasts.

Low-size AuNPs can enter the tumor cancer cells and accumulate there.^65^ Nandhini *et al.* reported a cytotoxicity property of AuNPs toward the HepG2 cell line, mediated by the production of ROS, reducing the expression of proliferating cell nuclear antigen, and dislocation of cytochrome *c* into the cytoplasm.^66^ Al-Radadi indicated an IC50 of 23 µg/mL by the biogenic AuNPs towered HepG2 cell line,^67^ which is approximately the current observed IC50 (53.45 µg/mL). There are multiple factors responsible for the cytotoxicity of AuNPs, including the capping agent.^68^ When broccoli phytochemicals became capping agents for AuNPs, they might tune the cytotoxic properties of AuNPs. The capping agents from broccoli extract can enhance the cytotoxic effect of AuNPs against HepG2, where the extract was reported to have anticancer activity.^69^ Moreover, broccoli-derived biogenic AuNPs exhibited selective cytotoxicity against HepG2 cells compared to the HDF cell line (Fig. 9). Anadozie *et al*. reported biogenic AuNPs with selective anticancer cytotoxicity, with no toxic effect against the normal cell line (KMST-6).^70^ On the contrary, Keskin *et al.* reported that AuNPs exhibited a cytotoxic effect against HDF at concentrations greater than 25 µg/mL, but not against cancer cells (U118 and SK-OV-3), attributing this to the proliferative behavior of cancer cells.^71^

**Fig. 10:**
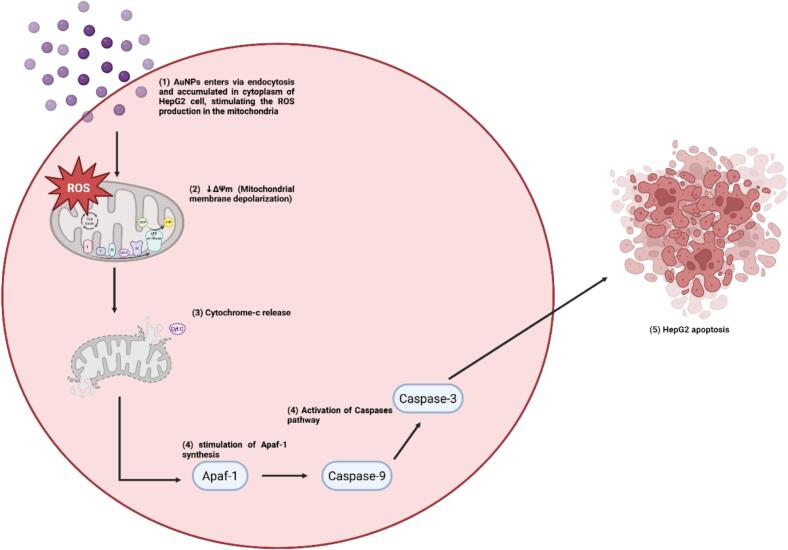
Proposed mitochondrial-mediated apoptosis pathway in HepG2 cells induced by broccoli-mediated AuNPs. After endocytic uptake, AuNPs increase intracellular ROS, leading to mitochondrial membrane depolarization (ΔΨm loss) and mitochondrial outer membrane permeabilization (MOMP). The release of cytochrome-c into the cytosol promotes apoptosome formation through Apaf-1, triggering caspase-9 activation, followed by activation of executioner caspase-3, ultimately resulting in apoptosis.

The enhanced cytotoxicity of broccoli-mediated AuNPs against HepG2 cells, compared to their mild effect on HDF cells, can be linked to differences in redox homeostasis, metabolic demand, and mitochondrial sensitivity between cancerous and normal cells. HepG2 cells possess intrinsically elevated basal ROS levels and more active mitochondrial metabolism,^72^, ^73^ making them more vulnerable to oxidative stress and apoptosis when exposed to AuNP-induced ROS production. After cellular uptake, mainly through endocytosis, AuNPs cause excessive ROS formation, disrupt mitochondrial membrane potential (ΔΨm)^26^, ^74^, and trigger cytochrome-c release, leading to the activation of caspase-dependent apoptosis pathways^75^. Elevated ROS also causes oxidative DNA damage and lipid peroxidation,^76^, ^77^, ^78^ speeding up cell death in HepG2 cells. Additionally, AuNPs can inhibit pro-survival signaling pathways (e.g., PI3K/AKT),^79^ further increasing the sensitivity of hepatocellular carcinoma cells to apoptosis (Fig. 10).

In contrast, HDF exhibits stronger antioxidant defenses and more stable mitochondrial function. Their lower basal ROS levels, combined with higher activity of enzymatic antioxidants such as SOD, CAT, and GSH-Px, enable them to resist oxidative changes caused by AuNPs, leading to minimal cytotoxicity.^30^, ^80^ Additionally, phytochemicals capping the biosynthesized AuNPs may provide a protective effect on normal cells by scavenging free radicals and regulating redox balance, thus reducing nonspecific toxicity.^9^, ^28^, ^81^ This dual behavior explains the selective cytotoxicity of green-synthesized AuNPs, which function as pro-oxidants in cancer cells while remaining largely biocompatible with normal tissue.

## 4 Conclusions

In this study, broccoli-mediated gold nanoparticles were successfully synthesized using a green approach and showed combined antioxidant, wound healing, and selective anticancer activities. The AuNPs enhanced wound closure and tissue regeneration, demonstrated by improved re-epithelialization and collagen deposition, and triggered lower inflammatory responses *in vivo*. They also exhibited selective toxicity toward HepG2 cells while remaining highly compatible with normal fibroblasts, highlighting the different sensitivities of cancer cells to oxidative and mitochondrial stress. Overall, these results suggest that broccoli-derived AuNPs have promising multifunctional therapeutic potential.

### 4.1 Study limitations

This study offers valuable insights into the antioxidant, wound-healing, and selective anticancer properties of broccoli-mediated gold nanoparticles; however, several limitations should be recognized. First, the mechanistic pathways behind the observed biological effects were inferred from literature and phenotypic results rather than confirmed through molecular or proteomic analyses. Second, while the *in vivo* wound model showed promising regenerative potential, it was limited to a small animal group and a short healing period, preventing conclusions about long-term efficacy or safety. Third, the cytotoxicity assessment was limited to a single cancer cell line (HepG2) and one normal cell type, which restricts the generalizability of the findings across broader cellular systems. Finally, dosage optimization, biodistribution, and systemic toxicity of the nanoparticles were not evaluated and should be addressed in future studies to support potential clinical translation better.

Ethical approval.

This work was approved by the ethics committee at the College of Science, 10.13039/501100015317Mustansiriyah University (Ref. BCSMU/0124/0001C on 10/01/2024).

## CRediT authorship contribution statement

**Yasser M. Taay:** Conceptualization, Methodology, Validation, Formal analysis, Investigation, Resources, Data Curation, Writing – original draft, Writing – review & editing, Visualization, Project administration, Funding acquisition. **Mustafa Taha Mohammed:** Writing – review & editing, Writing – original draft, Visualization, Supervision, Conceptualization. **Ali Hussain Alwan:** Writing – review & editing, Writing – original draft, Visualization, Supervision, Methodology. **Ahmad Hussein Ismail:** Supervision.

## Declaration of competing interest

The authors declare that they have no known competing financial interests or personal relationships that could have appeared to influence the work reported in this paper.
